# A tailored e-learning gives long-term changes in determinants of GPs’ benzodiazepines prescribing: a pretest-posttest study with self-report assessments

**DOI:** 10.1080/02813432.2019.1663591

**Published:** 2019-09-18

**Authors:** Hanne Creupelandt, Sibyl Anthierens, Hilde Habraken, Coral Sirdifield, Aloysius Niroshan Siriwardena, Thierry Christiaens

**Affiliations:** a Department of Public Health and Primary Care, Ghent University, Ghent, Belgium;; b Department of Primary Health Care and Interdisciplinary Care, University of Antwerp, Antwerp, Belgium;; c BCFI Vzw, p.a. Federaal Agentschap Voor Geneesmiddelen en Gezondheidsproducten Fagg, Brussels, Belgium;; d Community and Health Research Unit, School of Health and Social Care, University of Lincoln, Lincoln, UK;; e Clinical Pharmacology Research Unit, Heymans Institute of Pharmacology, Ghent University, Ghent, Belgium

**Keywords:** Benzodiazepine, general practitioner, family practice, continuing medical education, drug prescription, education

## Abstract

**Objective:** Despite guidelines and campaigns, general practitioners (GPs) continue to overprescribe benzodiazepines (BZDs). New approaches to improve prescribing are needed. Using behavior change techniques and tailoring interventions to user characteristics are vital to promote behavior change. This study evaluated the impact of a tailored e-learning module on factors known to determine BZD prescribing within GPs.

**Design:** A pretest-posttest study design with three self-report assessments concerning determinants of BZD prescribing: at baseline, immediately after the module (short term) and six months after completion (long term).

**Setting:** Flanders (Belgium)

**Intervention:** A tailored e-module that focuses on avoiding initial BZD prescriptions and using psychological interventions as an alternative.

**Subjects:** 244 GPs

**Main outcome measures:** Assessed determinants include GPs’ attitudes concerning treatment options, perceptions of the patient and self-efficacy beliefs. Readiness to adhere to prescribing guidelines was evaluated through assessing motivation, self-efficacy and implementability of non-pharmacological interventions.

**Results:** A significant and durable impact on determinants of BZD prescribing was observed. GPs underwent desirable changes in attitudes, perceptions and self-efficacy beliefs and these changes remained significant six months later.

**Conclusion:** Tailoring an e-intervention to target group characteristics appears to be successful in promoting behavioral change in experienced GPs. Significant and lasting changes were observed in determinants of prescribing BZDs.

**Key Points**
- A tailored e-intervention resulted in significant and long term changes in previously identified determinants of prescribing BZDs. The e-module resulted in a positive impact on GPs’ readiness to adhere to BZD prescribing guidance and the way they experience psychosocial consultations. Tailoring an e-intervention to target group characteristics appears to be successful in promoting behavioral change in experienced GPs.

## Introduction

Benzodiazepines (BZDs) are commonly used psychotropic drugs for treating conditions such as insomnia and anxiety in primary care, although their long-term use is associated with considerable adverse effects [1–3]. Despite guidance advocating use of non-pharmacological, psychological treatments first-line (1), and restricting BZDs to only short-term use if needed (2), numerous studies have shown that BZDs are still overprescribed and commonly used long-term [4–6].

General Practitioners (GPs) regard BZD prescribing as one of the most complex, demanding and uncomfortable tasks in their clinical work [7] but they continue to prescribe these drugs frequently and many prescriptions are even issued without the GP seeing the patient [8]. Reviews identify a variety of reasons for inconsistent BZD prescribing in primary care [9]. The main concern for GPs is to help the patient: GPs try to manage the tension between minimizing prescribing and their responsibility to help ‘deserving’ patients [7,10].

Sirdifield et al. [9] developed an explanatory model of processes underlying current prescribing practices of BZDs in primary care. This model can be used to support and evaluate interventions to improve adherence to BZD prescribing guidance. The model emphasizes that prescribing BZDs is a behavioral outcome determined by several factors. Interventions should therefore not merely focus on acquiring knowledge about BZDs or on non-pharmacological alternatives but should also address other determinants contributing to inconsistent prescribing practices. Thus, training programs should also focus on ambivalent attitudes and perceptions, as they are important determinants of inconsistent prescribing strategies [11].

Educational interventions for Continuing Medical Education (CME) typically focus on acquiring knowledge about correct prescribing [12] and are seldom attuned to the psychological dynamics of GPs. Using behavior change techniques is vital however, when promoting behavior change [13,14].

Implementation science literature emphasizes the importance of understanding key determinants and a need for multifaceted and tailored strategies. Interventions tailored to prospectively identified barriers are more likely to improve professional practice than dissemination of guidelines or educational materials alone [15].

Tailoring an intervention concerning BZD prescribing to target group characteristics was successful in promoting behavioral change in inexperienced GPs undertaking vocational training [16], but the impact of a tailored intervention on experienced and practicing GPs remains unknown.

This study evaluates the impact of a tailored e-module on the readiness of experienced GPs to adhere to BZD prescribing guidance: advocating the use of non-pharmacological treatments first-line, and using BZDs only short-term and if needed.

## Material and methods

### Participants and process

Over a five year period (2012–2016), Flemish GPs had the opportunity to participate in a free e-module developed by a multidisciplinary team at Ghent University. The e-module was promoted on the website of the Flemish professional organization for GPs (Domus Medica). GPs who gave informed consent for participating in the study were asked to complete a self-report assessment when starting and ending the e-learning module. Six months after completion, participants received an email invitation to complete a post-intervention questionnaire. Non-responders were sent up to four reminders. All data were anonymized before analysis.

### Context

This study was conducted in Flanders (the Dutch-speaking part of Belgium). Belgium has an accessible health system, but mental health and mental healthcare indicators are alarming [17]. Relevant within the context of BZD prescribing is that in Belgium there is fee-for-service payment and there is no patient list (with a free choice of GP). BZD consumption in Belgium is very high: 13% of the adult population had used BZD in the two weeks preceding a national health interview survey. Among females aged 75+, use rises to 40% [18]. This despite the fact that in the past decennium several campaigns have been organized by the Federal Government to address this issue.

### Intervention

The intervention (Additional File) focused primarily on avoiding initial BZD prescriptions and using psychological interventions [19–23] as an alternative. The intervention was a web-based and tailored program: it sought to address to GPs’ ambivalent attitudes and perceptions, which are known to determine their prescribing practices [9] and not merely focus on GPs acquiring knowledge. The e-module drew on the theory of Self-Determination (SDT [24], which provides both theoretical grounds and practical guidelines to create motivating learning environments [24–26]. According to SDT [24], the e-module aimed to fulfil basic psychological needs for autonomy, competence, and relatedness of participants.

### Outcome measures

The intervention was evaluated for its impact on previously identified determinants and readiness to adhere to prescribing guidelines [9]. Assessed determinants included attitudes concerning treatment options, perceptions of the patient and self-efficacy beliefs (Table 1). Readiness to adhere to prescribing guidelines was evaluated by assessing motivation, self-efficacy (Table 2) and implementability of non-pharmacological interventions (Table 3) (Additional File).

**Table 1.** Impact on GPs’ Psychological Determinants of BZD prescribing (*n* = 244) at baseline, when ending the module (short term) and six months later (long term).

	Strongly disagree (%)	Disagree (%)	No opinion (%)	Agree (%)	Strongly agree (%)	Wilcoxon *Z*	*p* Value
GPs’ attitudes concerning treatment options, determining BZD prescribing							
1. The advantages of sleep medication outweigh the disadvantages.							
Baseline	28.4	47.7	17.3	6.6	0		
Short term	45.2	38.2	10.8	3.3	2.5	−4.055	<.001
Long term	42.2	38.4	11.4	5.9	2.1	−2.853	.004
2. There are no non-drug alternatives for sleep problems that are as effective as drugs.							
Baseline	19.3	45.1	19.7	13.5	2.5		
Short term	36.3	42.1	11.3	7.9	2.5	−4.390	.000
Long term	31.1	45.4	15.1	7.6	.8	−4.487	.000
3. I don’t have time to treat sleep problems using non-drug therapies.							
Baseline	17.3	45.7	20.2	16.5	.4		
Short term	15.4	44.8	24.5	14.5	.8	−.645	.519
Long term	18.9	42.4	21.4	15.1	2.1	−.080	.937
4. The non-medication treatment of sleep problems is the business of other professionals.							
Baseline	25.8	49.6	16	7	1.6		
Short term	23.3	52.1	16.7	6.3	1.7	−.068	.945
Long term	30.8	44.3	16.5	8.4	0	−.585	.559
5. Non-drug treatment of sleep problems needs to be supported with medication.							
Baseline	23.5	50.2	23	3.3	0		
Short term	33.3	50.4	12.9	2.9	.4	−3.301	.001
Long term	39.8	47.5	12.3	.4	0	−6.212	<.001
GPs’ perception of the patient, determining BZD prescribing							
6. If I do not prescribe medication to a patient with sleep problems, (s)he is dissatisfied.							
Baseline	5.3	32.4	23	37.7	1.2		
Short term	6.6	42.7	27	22.4	1.2	−3.893	<.001
Long term	9.2	36.6	27.3	24.4	2.5	−2.919	.004
7. It is difficult for a GP to motivate a patient with sleep problems to choose a non-medication. treatment.							
Baseline	4.5	16.9	9.5	53.7	15.3		
Short term	.8	32.9	14.6	46.3	5.4	−4.674	<.001
Long term	5.5	25.2	18.1	45	6.3	−4.592	<.001
GPs’ self-efficacy beliefs, determining BZD prescribing							
8. When I am not prescribing medication for sleep problems I feel like I am not empathic.							
Baseline	33.6	41.4	11.5	13.1	.4		
Short term	35.8	47.5	10.8	5.8	0	−2.657	.008
Long term	43.7	40.3	10.1	4.2	1.7	−3.180	.001
9. I have the expertise to use non-drug treatment for sleep problems.							
Baseline	6.6	38.1	34	20.5	.8		
Short term	2.1	12.1	29.2	53.8	2.9	−8.990	<.001
Long term	3.4	13.1	26.6	51.1	5.9	−7.851	<.001
10. I often feel overwhelmed when a patient presents with psychosocial problems.							
Baseline	27.9	46.7	12.7	12.3	.4		
Short term	25.3	52.7	13.3	8.3	.4	−.935	.350
Long term	27.4	45.1	17.7	8.4	1.3	−.266	.790

**Table 2.** GPs’ readiness to adhere to prescribing guidelines (*n* = 244) at baseline, when ending the intervention (short term) and more than six months later (long term).

Intention to change	selected by (%)	Mc Nemar χ_2_	*p* Value
1. I intend to prescribe less sleep medication within the next weeks (<one month).			
Baseline	12.7		
Short term	34	32.513	<.001
Made efforts to change			
2. I have tried in the past to prescribe less sleep medication			
Baseline	46.3		
Long term	59.8	27.534	<.001
Self-efficacy beliefs			
3. I intend to prescribe less sleep medication but don’t know how.			
Baseline	17.6		
Short term	1.7	35.220	<.001
Long term	.8	33.231	<.001
4. I am trying at the moment to prescribe less sleep medication but without success.			
Baseline	29.1		
Short term	14.5	22.667	<.001
Long term	7.1	36.125	<.001
5. I am trying at the moment to prescribe less sleep medication and have succeeded in doing so.			
Baseline	18.4		
Short term	46.1	51.247	<.001
Long term	56.9	72.640	<.001

**Table 3.** Implementability of 6 demonstrated alternative treatment strategies (*n* = 244) Perceived meaningfulness and usefulness of treatment strategies when ending the module (short term) and perceived usefulness and actual use more than six months later (long term).

	Short term		Long term	
	Meaningful (%)	Useful (%)	Useful (%)	Used (%)
1. ICE model of communication				
strongly disagree / never used	0	0	2.1	14.5
disagree	1.6	3.7	5	
neutral. no opinion / rarely used	6.2	10.8	17.4	34.9
agree	42	50.6	49.2	
strongly agree / frequently used	50.2	34.9	26.4	50.6
1. Sleep hygiene education				
strongly disagree / never used	0	0	.4	3.3
disagree	.8	.8	.8	
neutral. no opinion / rarely used	2.5	2.5	5	28.9
agree	28.2	30	37.2	
strongly agree / frequently used	68.5	66.7	56.6	67.8
2. Stress-vulnerability model				
strongly disagree / never used	.4	.8	.8	18.9
disagree	3.3	5	4.6	
neutral. no opinion / rarely used	9.5	14.9	13.3	43.7
agree	48.1	43.6	44.6	
strongly agree / frequently used	38.6	35.7	36.7	37.4
3. Sleep wake diary				
strongly disagree / never used	.4	1.2	5.4	35.7
disagree	2.1	3.7	7.9	
neutral. no opinion / rarely used	6.6	19.9	24.5	53.5
agree	49.2	45.2	47.7	
strongly agree / frequently used	41.7	29.9	14.5	10.8
4. Stimulus control therapy				
strongly disagree / never used	0	.4	1.7	32.5
disagree	1.2	1.7	7.4	
neutral. no opinion / rarely used	7.1	13.2	28.9	50
agree	46.9	43	40.1	
strongly agree / frequently used	44.8	41.7	21.9	17.5
5. ABC model				
strongly disagree / never used	.4	1.2	2.9	47.1
disagree	5.4	10.7	14.2	
neutral. no opinion / rarely used	10.7	25.2	26.7	43.8
agree	54.1	44.6	43.3	
strongly agree / frequently used	29.3	18.2	12.9	7.1

### Assessments

Three self-report assessments took place: one pre-intervention (baseline) and two post-intervention assessments: immediately after completing the module (short-term) and more than six months after completion (long-term) (Additional file).

### Analysis

Effects of the intervention were identified within a pretest-posttest study design. Short-term effects were identified using the assessment undertaken when participants completed the module as posttest. An assessment was sent six months after completing the intervention to identify long-term effects. The impact of the intervention on psychological determinants was analyzed using the non-parametric Wilcoxon signed-rank test. Effects on readiness to adhere to guidelines was analyzed using the nonparametric McNemar Bowker test. All statistical analyses were performed SPSS version 24.

## Results

### Participants (n = 244)

During a five-year period (2012–2016) 722 (out of approximately 7500 registered) Flemish GPs used the e-module and completed a baseline self-report assessment. Of these, 51% (*n* = 371) also completed a self-report assessment when completing the e-module. Most participants (83%) reported having spent more than two hours on the e-module and most (75%) chose to spread the e-learning over time (as suggested), taking a median of 14 days.

244 GPs (34% of the initial 722 participants) also completed the long-term follow-up assessment, with a median of seven months between this assessment and the baseline assessment. Only participants (*n* = 244) who completed all three assessments were included in further analysis.

Table 4 shows gender and years of experience of the participants at all three assessments; only small differences were observed between different groups.

**Table 4.** Gender and years of experience of GPs who participated at the baseline assessment (*n* = 722), in survey 1 and 2 (*n* = 371) and of GPs who participated all 3 assessments (*n* = 244).

		Baseline assessment (*n* = 722)			Two assessments (*n* = 371)			Three assessments (*n* = 244)		
		Male (%)	Female (%)	Total (%)	Male (%)	Female (%)	Total (%)	Male (%)	Female (%)	Total (%)
<10 years (%)		15.1	34.3	49.4	16.3	38.5	54.8	15.2	41.4	56.6
10–20 years (%)		4.2	12	16.2	2.9	10.2	13.1	1.6	10.7	12.3
20–30 years (%)		8.9	6.4	15.2	8	5.1	13.1	8.6	5.3	13.9
>30 years (%)		16.1	3	19.1	16	2.9	19	13.1	4.1	17.2
Total (%)		44.2	55.8	100	43.3	56.7	100	38.5	61.5	100

Only participants (*n* = 244) who completed all three assessments were included in further analysis (marked pale grey).

### Determinants of BZD prescribing at baseline

Baseline characteristics show that many participating GPs (46.3%) had previously tried to prescribe less sleep medication, but only 18.4% succeeded in doing so (Table 2).

Consistent with these efforts, many participating GPs showed several desirable attitudes and self-efficacy beliefs at baseline (Table 1): for several items (1, 4, 5, 8, 10), more than 2/3 participants reported desirable attitudes and self-efficacy beliefs. Psychological determinants of BZD prescribing are considered ‘desirable’ when participants indicate ‘helping’ attitudes, perceptions or beliefs on the Likert scale. Not having an opinion is therefore considered a ‘non-desirable’ factor.

A significant number of participants reported attitudes that were barriers to adherence to BZD prescribing guidance at baseline: 35.7% questioned whether non-drug alternatives were as effective as medication and 37% were not sure they had enough time to use non-drug therapies (items 2, 3).

How our participants perceived sleepless patients also appeared to be a barrier to adherence to prescribing guidance. Few (37.7%) participants were convinced that they could satisfy a patient without a drug prescription (item 6). Furthermore, most participants (69%) agreed that motivating patients to choose a non-drug treatment was difficult (item 7). Concerning self-efficacy, at baseline only 21.3% of the participating GPs felt they had the expertise to use a non-drug treatment (item 9).

### Impact of the e-module on psychological determinants

The assessments showed a desirable, significant and durable impact of the intervention for many determinants of BZD prescribing (Table 1). In this paragraph, we focus on the long term impact of the module, but the (analogous) short term results are listed in the table as well.

First, the assessments showed enduring changes in GPs’ attitudes concerning treatment options: several months after the intervention, attitudes changed significantly. Participants disagreed more strongly with the statement that the advantages of BZDs outweigh their disadvantages (*Z* = −2.853, *p* = .004) and they judged more strongly that non-drug alternatives are as effective as BZDs (*Z* = −4.487, *p* = .000). Participants disagreed more strongly that non-drug treatments need to be supported with medication (*Z* = −6.212, *p* < .001). Neither the idea of lacking time to use non-drug interventions nor perceiving them as a business of other professionals were changed significantly (item 3–4).

In addition, the intervention significantly influenced the way GPs perceived patients with sleep problems. Months after the intervention and presumably after seeing many patients, participants were less convinced that it took a drug prescription to satisfy a patient (*Z* = −2.919, *p* = .004) and they agreed less that it was difficult to motivate patients to choose a non-drug treatment (*Z* = −4.592, *p* < .001).

Further, participants reported a significantly stronger sense of self-efficacy. Participants disagreed more strongly that they had to show empathy by prescribing (*Z* = −3.180, *p* = .001) and felt more strongly that they had the expertise to use non-pharmacological interventions (*Z* = −7.851, *p* < .001). The number of GPs who felt they had such expertise almost tripled: from 21.3% at baseline to 57% several months after the intervention.

The module did not have an impact on feeling overwhelmed when a patient presents with psychosocial problems, but few participants (12.7%) reported this barrier at baseline.

### Impact of the e-module on readiness to adhere to prescribing guidelines

The module showed significant and desirable effects on participants’ readiness to change their prescribing behavior (Table 2). Firstly, the intervention appeared to have a significant and positive effect on participants’ intentions and efforts to change their prescribing practices: on completing the module the number of participants intending to prescribe fewer BZDs almost tripled.

More relevant however, was the significant and enduring impact on participants’ self-efficacy beliefs. The small number (17.6%) of GPs who reported not to know how to meet their goal of prescribing fewer BZDs reduced to almost zero in the longer term. Furthermore, analyses showed a significant effect on the number of participants reporting success in minimizing BZD prescribing. When starting the module, only 18.4% reported success, while several months after completing the e-module, 56.9% reported successfully minimizing BZD prescribing: this number more than tripled.

At the time of ending the e-module, 95.8% of the participants evaluated the intervention as ‘meaningful’ and months after the intervention more than 85% stated that the module did change their prescribing practice.

Participants reported implementing several of the demonstrated, non-pharmacological interventions (Additional File) in their practice (Table 3): every approach included in the e-module was used and considered practically useful by most participants. In particular, the ‘ICE model of communication’ (50.6%) and the booklet on sleep hygiene education (67.8%) were frequently used. ‘The ABC model’ (Activating events – Beliefs – Consequences) [27] was the non-drug intervention which was least often implemented, but was nevertheless used by 50.9% of our GPs. The ‘stress-vulnerability model’, ‘the sleep wake diary’ and the booklet on stimulus control therapy were considered practically useful by most, although opinions were divided on these interventions.

## Discussion

### Principal findings

We documented a significant and lasting impact of a tailored e-module on determinants of BZD prescribing among practicing GPs: GPs underwent desirable changes in previously identified attitudes, perceptions and self-efficacy beliefs and these changes remained significant six months later. Along similar lines, readiness to adhere to prescribing guidelines was enhanced and GPs reported experimenting with several demonstrated, non-drug interventions within these complex consultations.

### Strengths and weaknesses

The intervention described is innovative in several ways. It is a web-based and GP-tailored intervention that is attuned to GPs’ ambivalent attitudes, perceptions, and self-efficacy beliefs. It focuses on avoiding initial BZD prescriptions and using psychological treatments as an alternative. Although the best way to avoid BZD dependence is by not initiating these drugs [7], few interventions focus on initial prescriptions. Secondly, we demonstrate that a theoretical and psychological understanding of underlying processes helps to guide the development of effective interventions.

Thirdly, our study highlights that e-learning can be used effectively to attune to GPs and modify their attitudes, perceptions and self-efficacy beliefs in the longer term.

Fourthly, we demonstrated educational interventions targeting health professionals can be evaluated by selecting relevant psychological determinants as outcome measures. Thinking beyond decreased BZD prescriptions, we were able to strengthen GP characteristics that are likely to lead to better prescribing practices.

Our study has some important limitations as well. Voluntary participation is unavoidable, but suggests a sense of responsibility for BZD prescribing practice among participants. Also, reasons for dropping out of our study remain unknown. Thus a selection bias must be taken into account, certainly since participants’ motivation for engaging in the intervention and assessments was probably linked to the questioned determinants.

Another important limitation concerns the validity and reliability of the assessments, which is a common challenge when tailoring implementation strategies [28]. No validated scales were used, rather items were constructed to assess specific determinants of prescribing BZDs and readiness to adhere guidelines. Therefore caution is required when interpreting the observed changes.

Furthermore, we were not able to register the impact of the e-module on rates of BZD prescribing. Although operationalizing improvement in prescribing by registering quantities of BZD prescriptions is questionable, measuring actual behavior (change) would have been valuable. We documented the effectiveness of the intervention through durable changes in several psychological determinants of prescribing BZD. Since people do not always do what they want to do or think they do, it is inevitable that there is a gap between psychological determinants, reported behavior and actual behavior.

### Comparison with existing literature

Changing the behavior of health professionals is challenging [14] and not to the least it remains a pain point how to enhance adherence to mental health clinical guidelines [29]. The field of implementation science stresses that evidence based practice must be complemented by evidence-based implementation. Yet, many questions remain how to effectively translate evidence into public health impact [28]. Reviews continue to pinpoint implementation strategies often fail to address key determinants and mechanisms of change are seldom mentioned or tested when evaluating implementation strategies [28].

Concerning BZD prescribing, the tale is no different. We found no literature on other tailored interventions that specify and test mechanisms of change within GPs’ BZD prescribing. The same e-intervention appeared to be successful in promoting behavioral change in inexperienced GPs undertaking vocational training [16]. This study indicates it is meaningful in experienced GPs as well.

The e-intervention succeeded in motivating experienced GPs to actually implement several non-pharmacological interventions within their consultations. It is peculiar that implementing these non-pharmacological interventions did not highlight barriers such as lacking time for non-drug interventions or perceiving these to be the task of other professionals within GPs. This is surprising, since these are barriers to adhering prescribing guidelines often cited in literature [9].

### Implications

Although many questions remain, it is encouraging that an attuned e-intervention succeeded in changing something within experienced GPs on the longer term. Further research is needed to explore whether this could help to resolve the deadlock concerning BZD overprescription.

Since educational interventions that are obligatory tend to achieve a larger reduction in BZD prescribing [12], this could guide further research. Also, investigating which mechanisms of change (e.g. user characteristics) are responsible for the observed effectiveness could help to refine future interventions.

## Supplementary Material

Supplemental Material
