# Supplementary material for: A tailored e-learning gives long-term changes in determinants of GPs’ benzodiazepines prescribing: a pretest-posttest study with self-report assessments
Source: Scand J Prim Health Care. 2019 Sep 18;37(4):418–25. doi: 10.1080/02813432.2019.1663591 (PMC6883414; doi:10.1080/02813432.2019.1663591)
Supplement: Supplemental Material [file IPRI_A_1663591_SM4044.docx]

**Additional file: Method**

**Intervention: E-module concerning prescribing BZDs**

Avoiding initial BZD prescription and using alternative, non-pharmacological treatment strategies for patients suffering insomnia was the core focus of the E-intervention. Specifically, the module aspired to address to GPs’ ambivalent attitudes and perceptions, which are known to determine their prescribing practices [9] rather than merely focusing on GPs acquiring knowledge.

The E-module could be accessed online and participants could choose freely whether they completed the module at one sitting or in several stages. Though some suggestions were offered by the authors, participants were also given the autonomy to choose the order and amount of time they spent on items and chapters. The module was developed in a familiar format for GPs, creating a feeling of relatedness: participants met several virtual patients who were sitting in the waiting room of a general medical practice. Through video-consultations, trainees were shown the pitfalls of BZD prescriptions and how non-pharmacological alternatives could be used within the consultation room. The E-module provided training in enhanced communication skills. GPs were also provided with several tools and documents that could support them.

- The ‘ICE model of communication’ encourages GPs to explore patients ideas, concerns, and expectations about diagnosis and/or treatment (the ICE acronym) and exemplifies a patient-centered approach [19].
- ‘Sleep hygiene education’ refers to educating patients about healthy sleep behaviors and sleep-conducive environmental conditions [20].
- The ‘stress-vulnerability model’ (diathesis–stress model) suggests that people inherit a predisposition to mental illness, but this ‘vulnerability’ in itself is not sufficient to manifest the disorder and requires interaction with bio-psychosocial stressors [21].
- A ‘sleep wake diary’ is an assessment tool to explore patients’ sleep/wake patterns. This registration allows GP and patient to tailor interventions such as ‘sleep hygiene education’ or ‘stimulus control therapy’.
- ‘Stimulus control therapy’ is a classical conditioning approach to re-associating the bed and bedroom with successful sleep attempts [20].
- The ‘ABC Model’ can be used to educate patients about the relationship between thoughts, emotions and behavior, which is an important part of therapeutic change. The model can be used as a registration tool, inviting patients to record sequences of events in terms of ‘Activating events’, ‘Beliefs’ and’ Consequences’ [27].

To encourage reflection, video-consultations were shown in several stages and participants were frequently asked how and why they would act themselves in those particular situations. According to SDT [24], the E-module aimed to fulfil basic psychological needs for autonomy, competence, and relatedness of participants. Rather than telling participants what to do, the module gave participants a chance to experience the consequences of their choices as this enhances a feeling of control, competence and autonomy.

Scientific knowledge and guidelines concerning BZD prescribing was integrated and referred to within the module. However this was not the core material of the E-module, but was stored within a virtual ‘library’, available for participants whenever they wanted to consult some literature.

**Outcome measures**

***Selection of outcome measures***The intervention was evaluated for its impact on psychological determinants and readiness to adhere to prescribing guidelines. Assessed psychological determinants included GPs’ attitudes concerning treatment options and GPs’ perceptions of the patient, as described in the explanatory model of Sirdifield et al. [9]. This model emphasizes how prescribing BZDs is a behavioral outcome determined by several factors (GPs’ attitudes towards interventions, GPs’ perception of the patient, and GPs’ sense of responsibility). Since all trainees participated on a voluntary basis, the third determinant of the explanatory model (sense of responsibility for BZD prescribing practice) was considered present and was not assessed. Based on the findings that GPs appear to use BZDs to deal with their feelings of helplessness and uncertainty concerning both the doctor-patient relationship and non-drug alternatives for BZDs [7], items assessing GPs’ self-efficacy beliefs (beliefs about capabilities, a psychological determinant of behavioral intentions [22] were added to the self-report assessments.

Readiness to adhere to prescribing guidelines was evaluated by assessing motivation, self-efficacy and implementability of non-pharmacological interventions. Based on the transtheoretical model (TTM), a frequently used biopsychosocial model to conceptualize the process of intentional behavior change [23], participants were asked to declare whether they intended to make a change, had actually made efforts to change their prescribing practice and state self-efficacy beliefs concerning their ability to change their prescribing practice.
To assess the implementability of alternative, non-pharmacological treatment strategies, participants evaluated non-pharmacological interventions on their meaningfulness and practical usefulness and declared whether they had actually used the listed treatment strategies in their practice.

***Procedure***Three online self-report assessments took place: one pre-intervention (baseline) and two post-intervention assessments: immediately after completing the module (short-term impact) and more than six months after completion (long-term impact). All three self-report assessments comprised the same 10 items evaluating psychological determinants of BZD overprescribing in primary care (Table 1). All these items were rated agree/disagree on a 5-point Likert scale.

Additionally, items assessing participants’ readiness to adhere to BZD prescribing guidelines (Table 2) were added to the three assessments. At each assessment, participants were asked to make a selection of statements that they agreed with (a ‘select all that apply’-questionnaire), in order to determine where they were situated on the continuum between ‘not prepared to prescribe less’ and ‘already prescribing little sleep medication’. In order to enhance reliability, opposite items were included.
‘Intention to make a change’ was assessed at baseline and when completing the module (short-time impact). Self-perceived ‘efforts to change’ were assessed at baseline and six months after completing the module (long-term impact). Items assessing self-efficacy beliefs concerning one’s ability to change were added at all three assessments.

The two post-intervention assessments comprised items assessing the implementability of six alternative treatment strategies (Table 3). After completing the module, participants evaluated these psychological interventions on their meaningfulness and practical usefulness using a 5-point Likert scale. Six months after ending the module, the non-drug treatment strategies were evaluated on their practical usefulness (again using a 5-point Likert scale) where participants were asked whether they ‘never’, ‘rarely’ or ‘frequently’ used the listed treatment strategies in their practice. In the last questionnaire – administered six months after ending the module - participants rated on a 5-point Likert scale whether they felt the E-intervention had changed their practice.
To consider validity, all items were discussed with an interdisciplinary expert group (GPs, psychologists and a sociologist) to evaluate the content validity of the questionnaire.
